# Effect of Corrosion on the Natural and Whirl Frequencies of a Functionally Graded Rotor-Bearing System Subjected to Thermal Gradients

**DOI:** 10.3390/ma13204546

**Published:** 2020-10-13

**Authors:** Prabhakar Sathujoda, Bharath Obalareddy, Aneesh Batchu, Giacomo Canale, Angelo Maligno, Roberto Citarella

**Affiliations:** 1 Department of Mechanical and Aerospace Engineering, Bennett University, Greater Noida 201310, India; bo2559@bennett.edu.in (B.O.); ba8527@bennett.edu.in (A.B.); 2 Institute for Innovation in Sustainable Engineering, University of Derby, Derby DE1 3HD, UK; G.Canale@derby.ac.uk (G.C.); A.Maligno@derby.ac.uk (A.M.); 3 Department of Industrial Engineering, University of Salerno, 84084 Salerno, Italy; rcitarella@unisa.it

**Keywords:** functionally graded materials, corrosion, Timoshenko beam theory, finite element method, rotor-bearing systems

## Abstract

Corrosion causes a loss of material resulting in the reduction of mass and stiffness of a component, which consequently affects the dynamic characteristics of any system. Fundamental frequency analysis of a corroded functionally graded (FG) rotor system, using the finite element method based on the Timoshenko beam theory, was investigated in the present paper. The functionally graded shaft consisting of an inner metallic core and an outer ceramic layer was considered with the radial gradation of material properties based on the power law. Nonlinear temperature distribution (NLTD) based on the Fourier law of heat conduction was used to simulate the thermal gradient through the cross-section of the FG rotor. The finite element formulation for a functionally graded shaft with a corrosion defect was developed and the dynamic characteristics were investigated, which is the novelty of the present work. The corrosion parameters such as length, depth and position of the corrosion defect in the shaft were varied and a parametric study was performed to investigate changes in the natural and whirl frequencies. An analysis was carried out for different power indexes and temperature gradients of the functionally graded shaft. The effects of corrosion were analysed and important conclusions are drawn from the investigations.

## 1. Introduction

Functionally graded materials (FGMs) are an advanced type of composite material that has gained practical importance in recent years. FGMs are composed of metals, ceramics and polymers whose volume fractions vary in the desired directions based on material laws such as power and exponential and sigmoid laws. They are classified into ceramic–ceramic, metal–metal, metal–ceramic, ceramic–polymer and so on. Metal–ceramics are widely used due to their ability to withstand high temperatures, good mechanical performance and high specific strength and fracture toughness. They have been used for rocket engine components, aerospace structures, turbine blades, etc., and have found applications in aerospace, aircraft, automotive, biomedical, power, energy, electronics and chemical industries, among others. The general idea of functionally graded materials was proposed in 1972 for ceramics and polymers, inspired by the material structures of bones, teeth and bamboo trees. The concept of functionally graded materials was developed and the term was coined for the first time by Japanese scientists in the mid-1980s for aerospace applications. They were used as super resistant materials to reduce the generation of thermal stress and improve thermal resistivity in the propulsion systems of spacecraft. Since then, functionally graded materials have gained much importance around the world for various engineering applications [1,2,3,4,5]. Given the wide range of applications, FGMs, when used in harsh environmental conditions at elevated temperatures for long durations of time, can undergo corrosion.

Ever since the concept of functionally graded materials was introduced, many researchers have studied their behaviour and performance. Pindera and Dunn [6] developed a higher-order theory for FG plates subjected to through-thickness thermal gradients and compared the stress fields with finite element (FE) analysis. Aboudi et al. [7] generalised the higher-order theory based on Cartesian coordinates for FG materials. Recent works on the modelling and isogeometric analysis of functionally graded material structures are reported in the literature [8,9]. Few works have been reported in the literature on the vibration analysis of functionally graded beams. Aydogdu and Taskin [10] studied the free vibration analysis of a simply supported FG beam using Hamilton’s principle for different higher-order shear deformation and classical beam theories. Simsek [11] analysed the natural frequencies of functionally graded beams for different boundary conditions based on various beam theories. Alshorbagy et al. [12], using the finite element method, investigated the free vibration characteristics of an FG beam based on the Euler–Bernoulli beam theory.

Functionally graded materials (FGMs) are known for their ability to withstand extremely high-temperature environments. FGMs are also considered potential structural materials for future high-speed spacecraft. Reddy and Chin [13] studied the dynamic thermoelastic response of functionally graded cylinders and plates using the finite element method, including thermomechanical coupling in the formulation. Kawasaki and Watanabe [14] simulated a real environment using a H_2_/O_2_ combustion flame to study the thermal fracture behaviour of FG materials. Lanhe [15] derived stability and equilibrium equations based on the first shear deformation theory for a functionally graded thick rectangular beam under thermal loads to calculate the buckling temperature.

However, limited works are reported in the literature on the vibration analysis of functionally graded rotor-bearing systems. Gayen and Roy [16] carried out a vibration and stability analysis of an FG rotor-bearing system using a three-node finite beam element based on Timoshenko beam theory (TBT). Rao and Roy [17] carried out a dynamic analysis of a functionally graded rotating shaft system using the Timoshenko beam theory. Different analyses have been carried out including the Campbell diagram, stability speed limit and damping ratio. Bose and Sathujoda [18] performed a natural frequency analysis of a functionally graded rotor system using a three-dimensional finite element model developed using ANSYS (ANSYS 18.0, ANSYS, Canonsburg, PA, USA). Furthermore, Bose and Sathujoda [19] extended this work and studied the effects of thermal gradients on the vibration characteristics of an FG rotor-bearing system.

The effects of various defects on the vibration characteristics of structures and rotor systems have always been of great significance among researchers, and related works are reported in the literature. Gillichet al. [20] developed two kinds of mathematical relations for predicting frequency changes due to the two main effects of corrosion—loss of mass and decrease in stiffness. Shekar and Prabhu [21] studied the effect of coupling misalignments on the vibration characteristics of a rotor-bearing system. Prabhakar et al. [22] studied a method to detect cracks in a rotor-bearing system by measuring mechanical impedance. However, very limited works are available on the effects of defects present in functionally graded material systems. Gayen et al. [23] carried out a finite-element-based dynamic analysis of a functionally graded (FG) shaft with a transverse crack using a two-node Timoshenko beam element and considered the effects of translational and rotary inertia, transverse shear deformations and gyroscopic moments. Wattanasakulpong and Ungbhakorn [24] performed a linear and nonlinear vibration analysis of FG beams with porosities, elastically restrained by the ends, using the differential transformation method. Ferreira et al. [25] investigated the corrosion behaviour of Al/Al_3_Ti and Al/Al_3_Zr functionally graded materials formed by the centrifugal casting method and studied the influence of intermetallic platelets on corrosion behaviour. Musbah et al. [26] investigated the corrosion behaviour of Ti-B4C/CNF functionally graded materials produced in three layers by traditional cold compressing and sintering methods using the potentiodynamic method. Malinina et al. [27], using a high-velocity oxygen fuel spraying technique, carried out a comparative study of the corrosion resistance of FG (alumina-NiCr) and homogeneous environmental barrier coatings on steel substrates. However, to the best of the authors’ knowledge, works on the effects of corrosion on the vibration characteristics of functionally graded rotor systems are rarely reported in the literature.

Since most of the rotors operate at a constant speed and accelerate or decelerate at a constant angular acceleration or deceleration, uniform corrosion is possible especially when the shaft is exposed to a harsh corrosive environment over a long period at elevated temperatures. In this context, this paper investigates the effects of uniform corrosion on the natural and whirl frequencies of an FG rotor-bearing system using the finite element method based on the Timoshenko beam theory. A parametric study on the effect of corrosion was performed by varying depth, length and position of the corrosion defect. The study was performed for different power law indexes of material distributions in the FG shaft and temperature gradients in the shaft.

## 2. Material Modelling

A functionally graded shaft made up of metal and ceramic was considered for the present analysis. The shaft was subjected to thermal loads due to which the material temperature of the shaft varied nonlinearly in the radial direction. The material properties of the FG shaft depend on the temperature and metal–ceramic volume fractions and vary in the radial direction based on the power law. The temperature distribution of the shaft along the radial direction, the temperature-dependent material properties and the material gradation of the FG shaft are presented in the following subsections.

### 2.1. Nonlinear Temperature Distribution

The temperature distribution of the shaft varies along the radial direction and follows a nonlinear distribution *T*(*r*). It is obtained by solving a steady-state one-dimensional Fourier heat conduction equation with no heat generation, which is given in Equation (1).

(1) $$\frac{d}{dr}\left[K\left(r\right)\frac{dT}{dr}\right]=0$$

where the inner surface temperature is $T_{i}$ at the inner surface radius $r=R_{i}$, the outer surface temperature is $T_{o}$ at the outer surface radius $r=R_{o}$ and K(r)  is the thermal conductivity of the shaft at the radius  r. Solving Equation (1), the temperature distribution is given by [13] and is expressed as shown in Equation (2).

(2) $$T\left(r\right)=T_{i}+\left(T_{o}-T_{i}\right)\left[\frac{\sum_{j=0}^{5}\left\{\frac{{\left(-1\right)}^{j}}{jk+1}{\left(\frac{K_{oi}}{K_{i}}\right)}^{j\;}{\left(\frac{r-R_{i}}{R_{o}-R_{i}}\right)}^{jk+1}\right\}}{\frac{{\left(-1\right)}^{j}}{jk+1}{\left(\frac{K_{oi}}{K_{i}}\right)}^{j\;}}\right]$$

### 2.2. Temperature Dependence of Material Properties

The thermoelastic material properties of the metal and ceramic constituents used in the FG shaft are dependent on temperature. These properties follow a nonlinear relation with the material temperature. This relation given by Touloukian [28] and can be expressed as

(3) $$P\left(T\right)=\;P_{0}\left(P_{-1}T^{-1}+1+\;P_{1}T+\;P_{2}T^{2}+\;P_{3}T^{3}\right)$$

where *T* is the temperature in Kelvins, *P* is the property of the material and *P*_0_, *P*_−1_, *P*_1_, *P*_2_ and *P*_3_ are the temperature coefficients. These coefficients vary for different material constituents and properties. They are given by Reddy and Chin [13] and are tabulated in Table 1.

In the presented work, Young’s modulus, Poisson’s ratio and the thermal conductivity are temperature dependent. The mass densities of steel and ZrO_2_ were taken as 8166 kg/m^3^ and 5700 kg/m^3^, respectively, and were assumed to be independent of temperature.

### 2.3. Material Properties Gradation of the FG Shaft

**Figure 1:**
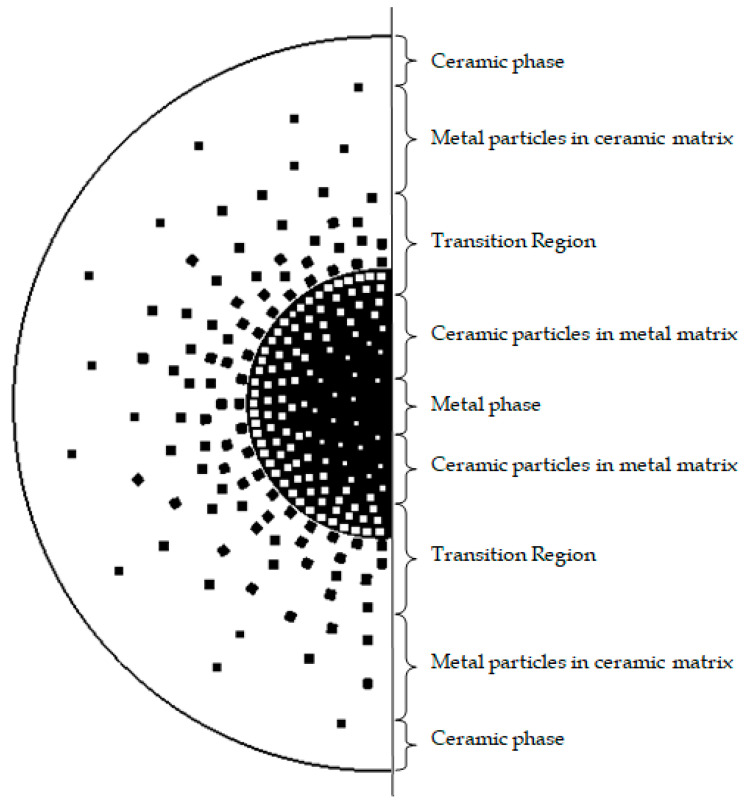
Material gradation of metal and ceramic phases along the radial direction of the shaft shown in the cross-sectional area of the shaft.

The FG shaft consists of an outer ceramic-rich region and an inner metal-rich region. The gradation of materials occurs between these layers as shown in Figure 1. To obtain a smooth variation of properties, the gradation is controlled using material laws. Researchers have reported several gradation schemes such as the power law, exponential law and sigmoid law gradations. However, the power law is used in most applications and also in the present work. The material property gradation of an FGM shaft is obtained by varying the volume fractions of metal and ceramic in the radial direction of the shaft. According to the rule of mixtures of effective material properties [24], *P* can be expressed as:

(4) $$P\left(r,T\right)=P_{m}\left(T\right)V_{m}\left(r\right)+P_{c\;}\left(T\right)V_{c}\left(r\right)$$

where $P_{m}$ and $P_{c\;}$ are the material properties and $V_{m}$ and $V_{c}$ are the corresponding volume fractions of metal and ceramic, respectively, at any given FG layer. The volume fraction of the metal and ceramic layers are related as given in Equation (5).

(5) $$V_{m}\left(r\right)+V_{c}\left(r\right)=1$$

The volume fraction of the ceramic constituent varying along the radial direction of the shaft given by the power law can be expressed as:

(6) $$V_{c}\left(r\right)={\left[\frac{\;r-R_{i}}{\;R_{o}-R_{i}}\right]}^{k}$$

Solving Equations (4)–(6), we get the effective material property at any given layer of the shaft as in Equation (7).

(7) $$P\left(r,T\right)=\;P_{m}\left(T\right)+\;\left(P_{c}\left(T\right)-P_{m}\left(T\right)\right){\left[\frac{\;r-R_{i}}{\;R_{o}-R_{i}}\right]}^{k}$$

## 3. Finite Element Formulation

**Figure 2:**
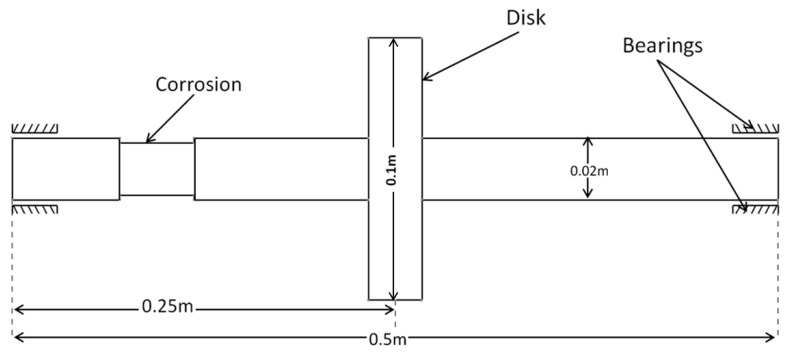
Functionally graded rotor-bearing system with a corrosion defect.

Finite element formulations were developed for the Jeffcott FG rotor-bearing system with the FG shaft with a corrosion defect and subjected to a thermal load as shown in Figure 2. This includes finite element formulations for the FG shaft elements with and without the corrosion defect, a steel disc and with isotropic bearings at its ends. These are presented step by step in the following subsections, respectively.

### 3.1. Corroded FG Shaft Elements

**Figure 3:**
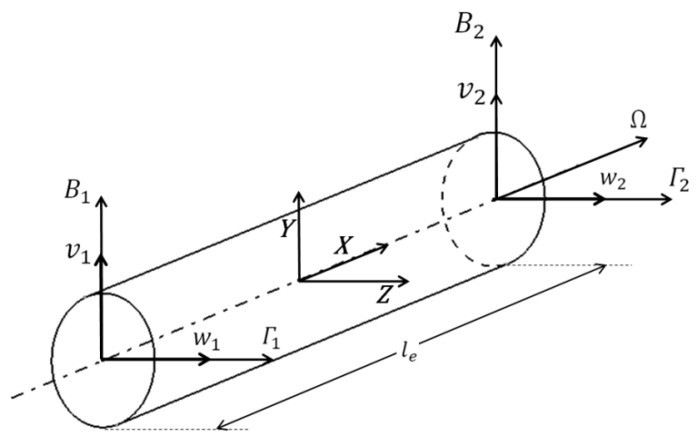
Functionally graded shaft element and its degrees of freedom.

The finite element formulations of the FG shaft were developed using Timoshenko beam elements with two nodes with four degrees of freedom per node, two translational (v, w) and two rotational (Β, Γ), where the effects of translational and rotary inertia, transverse shear deformations and gyroscopic moments were considered. As analysis was performed for bending natural frequencies, which are appropriate in the case of a Jeffcott rotor supported on the linear bearings in transverse directions, the axial translational degree of freedom was not considered. Figure 3 shows the FG shaft element with nodal degrees of freedom. The shaft was divided into finite beam elements of length ($l_{e}$). The Timoshenko beam element matrices for a homogenous shaft were first developed by Nelson [29] using Hamilton’s extended principle with energy and work functions. The equation of motion in matrix form for a finite rotating shaft element can be expressed as:

(8) $$\left(\left[M^{e}\right]+\left[N^{e}\right]\right)\left\{{\ddot{q}}^{e}\right\}-\Omega \left[G^{e}\right]\left\{{\dot{q}}^{e}\right\}+\left[K^{e}\right]\left\{q^{e}\right\}=\left\{Q^{e}\right\}$$

The obtained element matrices were developed by Gayen [23] for FG shaft elements. In the present work, the element matrices were developed for corroded FG shaft elements. The corrosion defect is introduced into the FG shaft element through the removal of mass on the circumference of the FG shaft element, hence reducing the radius of the shaft. The reduction in the radius of the FG shaft element is denoted as the corrosion depth  (d). The matrices for the corroded FG shaft element with a Young’s modulus  E(r,T), Poisson’s ratio ν(r,T), rigidity modulus G(r,T), shear factor κ(r,T), density ρ(r), mass per unit length m, diametric moment per unit length $I_{D}$, polar moment per unit length $I_{P}$, area of cross-section A and area moment of inertia I are as follows:

Elemental stiffness matrix:

(9) $$\left[K^{e}\right]=\overset{l_{e}}{\underset{0}{\int }}EI{\left[\psi^{\prime \prime }\right]}^{T}\left[\psi^{\prime \prime }\right]dyds+\overset{l_{e}}{\underset{0}{\int }}\kappa GA{\left[\psi^{\prime }\right]}^{T}\left[\psi^{\prime }\right]dyds\;EI=\overset{\;R_{o}-d}{\underset{R_{i}}{\int }}2\pi E\left(r,T\right)r^{3}dr\;\kappa GA=\overset{\;R_{o}-d}{\underset{R_{i}}{\int }}2\pi r\kappa \left(r,T\right)G\left(r,\;T\right)dr$$

Elemental translation mass matrix:

(10) $$\left[M^{e}\right]=\overset{l_{e}}{\underset{0}{\int }}m{\left[\psi \right]}^{T}\left[\psi \right]ds\;m=\overset{\;R_{o}-d}{\underset{R_{i}}{\int }}2\pi \rho \left(r\right)r^{3}dr$$

Elemental rotation mass matrix:

(11) $$\left[N^{e}\right]=\overset{l_{e}}{\underset{0}{\int }}I_{D}{\left[\varphi \right]}^{T}\left[\varphi \right]ds\;I_{D}=\overset{\;R_{o}-d}{\underset{R_{i}}{\int }}\pi \rho \left(r\right)r^{3}dr$$

Elemental gyroscopic matrix:

(12) $$\left[G^{e}\right]=\left[H^{e}\right]-{\left[H^{e}\right]}^{T}\;\left[H^{e}\right]=\overset{l_{e}}{\underset{0}{\int }}I_{P}{\left[\varphi_{w}\right]}^{T}\left[\varphi_{v}\right]ds\;I_{p}=\overset{\;R_{o}-d}{\underset{R_{i}}{\int }}2\pi \rho \left(r\right)r^{3}dr$$

In the case of uncorroded shaft elements, the integration limits are *Ri* to *Ro* in Equations (9)–(12). The variations of material properties are implemented by computing definite integrals of the properties across the shaft radius using a fixed-order Gaussian quadrature method.

Nodal displacement vector:

(13) $$\begin{array}{cc} \left\{q^{e}\right\}= & \left[\begin{array}{ccc} \begin{array}{ccc} v_{1} & w_{1} & {Β}_{1} \end{array} & \begin{array}{cc} \Gamma_{1} & v_{2} \end{array} & \begin{array}{ccc} w_{2} & {Β}_{2} & \Gamma_{2} \end{array} \end{array}\right] \end{array}$$

Spatial constraint matrix for translation shape functions:

(14) $$\left[\psi \left(s\right)\right]=\left[\begin{array}{cccccccc} \psi_{1} & 0 & 0 & \psi_{2} & \psi_{3} & 0 & 0 & \psi_{4}\\ 0 & \psi_{1} & -\psi_{2} & 0 & 0 & \psi_{3} & -\psi_{4} & 0 \end{array}\right]\;\psi_{j}\left(s\right)=\frac{1}{1+\Phi }\left[\alpha_{j}\left(s\right)+\Phi \beta_{j}\left(s\right)\right];\;j\;=\;1,\;2,\;3\;\mathrm{and}\;4;\mu =\frac{s}{l_{e}};\;\alpha_{1}=1-3\mu^{2}+2\mu^{3}\;\;\beta_{1}=1-\mu \;\alpha_{2}=l_{e}\mu \left(1-2\mu +\mu^{3}\right)\;\;\beta_{2}=l_{e}\mu \left(1-\mu \right)/2\;\alpha_{3}=\mu^{2}\left(3-2\mu \right)\;\;\beta_{3}=\mu \;\alpha_{4}=l_{e}\mu^{2}\left(-1+\mu \right)\;\;\beta_{4}=l_{e}\mu \left(-1+\mu \right)/2$$

Spatial constraint matrix for rotational shape functions:

(15) $$\left[\varphi \left(s\right)\right]=\left[\begin{array}{c} \left[\varphi_{v}\right]\\ \left[\varphi_{w}\right] \end{array}\right]=\left[\begin{array}{cccccccc} 0 & -\varphi_{1} & \varphi_{2} & 0 & 0 & -\varphi_{3} & \varphi_{4} & 0\\ \varphi_{1} & 0 & 0 & \varphi_{2} & \varphi_{3} & 0 & 0 & \varphi_{4} \end{array}\right]\;\varphi_{j}\left(s\right)=\frac{1}{1+\Phi }\left[\gamma_{j}\left(s\right)+\Phi \delta_{j}\left(s\right)\right];\;j\;=\;1,\;2,\;3\;\mathrm{and}\;4;\;\mu =\frac{s}{l_{e}}\;\gamma_{1}=\;\mu \left(6\mu -6\right)/l_{e}\;\;\delta_{1}=0\;\gamma_{2}=1-4\mu +3\mu^{2}\;\;\delta_{2}=1-\mu \;\gamma_{3}=\mu \left(-6\mu +6\right)/l_{e}\;\;\delta_{3}=0\;\gamma_{4}=\mu \left(3\mu -2\right)\;\;\delta_{4}=\mu$$

The transverse shear effect:

(16) $$\Phi =\frac{EI}{\kappa GA\times l_{e}{}^{2}}$$

**Figure 4:**
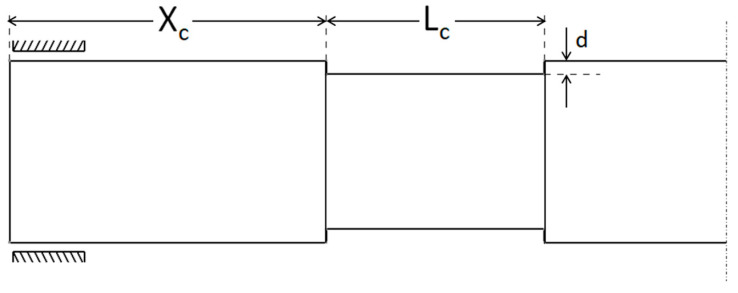
Various parameters of corrosion defect in the functionally graded rotor-bearing system.

Figure 4 shows the corrosion parameters used to study the effect of the corrosion defect. If m number of consecutive corroded FG shaft elements are placed after n FG shaft elements, then

The corrosion length:

(17) $$L_{c}=m\times l_{e}$$

The corrosion position from the left bearings:

(18) $$X_{c}=n\times l_{e}$$

### 3.2. Uniform Steel Disc

The FG shaft used for the present analysis has a uniform steel disc at its midspan. The disc has a mass ($m_{d}$), a diametral moment of inertia ($I_{d}$) and a polar moment of inertia ($I_{p}$). The translation mass matrix $\;\left[M^{d}\right]$, rotation mass matrix $\;\left[N^{d}\right]$, gyroscopic matrix $\;\left[G^{d}\right]$ and the governing equation of motion of the rigid disc are of the following form:

(19) $$\left(\left[M^{d}\right]+\left[N^{d}\right]\right)\left\{{\ddot{q}}^{d}\right\}-\Omega \left[G^{d}\right]\left\{{\dot{q}}^{d}\right\}+\left[K^{d}\right]\left\{q^{d}\right\}=\left\{Q^{d}\right\}\;\left\{q^{b}\right\}=\left\{\begin{array}{c} \begin{array}{c} v\\ w \end{array}\\ Β\\ \Gamma \end{array}\right\}\left[M^{d}\right]=\left[\begin{array}{cccc} m_{d} & 0 & 0 & 0\\ 0 & m_{d} & 0 & 0\\ 0 & 0 & 0 & 0\\ 0 & 0 & 0 & 0 \end{array}\right]\;\left[N^{d}\right]=\left[\begin{array}{cccc} 0 & 0 & 0 & 0\\ 0 & 0 & 0 & 0\\ 0 & 0 & I_{d} & 0\\ 0 & 0 & 0 & I_{d} \end{array}\right]\left[G^{d}\right]=\left[\begin{array}{cccc} 0 & 0 & 0 & 0\\ 0 & 0 & 0 & 0\\ 0 & 0 & 0 & -I_{p}\\ 0 & 0 & I_{p} & 0 \end{array}\right]$$

where {$q^{d}$} is the nodal displacement vector and $\left\{Q^{d}\right\}$ is the external force vector of the disc.

### 3.3. Linear Support Bearings

The equation of motion for isotropic bearings can be written as:

(20) $$\left[C^{b}\right]\left\{{\dot{q}}^{b}\right\}+\left[K^{b}\right]\left\{q^{b}\right\}=\left\{Q^{b}\right\}\;\left\{q^{b}\right\}=\left\{\begin{array}{c} v\\ w \end{array}\right\}\begin{array}{cc} , & \left[C^{b}\right] \end{array}=\left[\begin{array}{cc} C_{b} & 0\\ 0 & C_{b} \end{array}\right]\begin{array}{cc} , & \left[K^{b}\right] \end{array}=\left[\begin{array}{cc} K_{b} & 0\\ 0 & K_{b} \end{array}\right]$$

where [$C^{b}$] is the damping matrix, $\left[K^{b}\right]$ is the stiffness matrix, $\left\{{\dot{q}}^{b}\right\}$ is the nodal displacement vector and $\left\{Q^{b}\right\}$ is the external force vector.

### 3.4. System Equation of Motion and Solution Procedure

The equation of motion for the complete rotor-bearing system with no external force acting on the system can be expressed as:

(21) $$\left[M\right]\left\{\ddot{q}\right\}-\Omega \left[G\right]\left\{\dot{q}\right\}+\left[K\right]\left\{q\right\}=0$$

where [M] is the global mass matrix including translation and rotation mass matrices of all the shaft elements and the disc, [K] is the global stiffness matrix including stiffness matrices of all the shaft elements and the bearings, [G] is the global gyroscopic matrix including gyroscopic matrices of all the shaft elements and the disc and Ω is the spin speed of the rotor. {q} is the nodal displacement vector for the complete rotor-bearing system. The equation of motion for the complete rotor-bearing system can be rewritten as:

(22) $$A\dot{h}+\mathrm{Bh}=0\;A=\;\left[\begin{array}{cc} 0 & \left[M\right]\\ \left[M\right] & -\Omega \left[G\right] \end{array}\right],\;B=\left[\begin{array}{cc} -\left[M\right] & 0\\ 0 & \left[K\right] \end{array}\right],\;h=\left\{\begin{array}{c} \dot{q}\\ q \end{array}\right\}$$

The Eigenvalues obtained from the above equation are of the form:

(23) $$\lambda_{n}\left(\Omega \right)=\xi_{n}\left(\Omega \right)\;\pm \;i\omega_{n}\left(\Omega \right)$$

The (ξ) is damping constant and (ω) is the whirl frequency in  rad/s. At Ω = 0, (*ω*) is the natural frequency.

## 4. Validations

A finite element code was developed in Python (IDLE Python 3.6.1, 64 bit, Python Software Foundation, Wilmington, DE, USA) to solve the Eigenvalue problem outlined in Section 3 and compute the natural and whirl frequencies of the FG rotor system with and without corrosion. The developed FE formulation and Python code were validated with the published results to check the correctness of the formulations.

Validation was performed in two steps. In the first step, the dimensionless natural frequencies of a nonrotating simply supported homogeneous shaft were obtained using the developed code and compared with the existing results available for the same shaft to ensure the correctness of the mass and stiffness matrices. In the second step, the dimensionless natural frequencies obtained using the developed code for the nonrotating simply supported FG shaft were compared with previously published results to ensure the correctness of the FG modelling.

### 4.1. Natural Frequencies of Homogeneous Nonrotating Simply Supported Shaft

The dimensionless natural frequencies $\;\bar{\omega }\;({\bar{\omega }}^{4}=\rho_{ss}AL^{4}\omega^{2}/E_{ss}I)$ were obtained for the nonrotating simply supported steel shaft with $\;E=208\;\mathrm{GPa},\;\nu =0.3\;\mathrm{and}\;\rho =7800\;\mathrm{Kg}/m^{3}$ for different slenderness ratios SR=R/2L where *R* is the shaft radius and *L* is the shaft length. The computed results were compared with the published results of Gayen [26] and Nelson [29] and are tabulated in Table 2. It can be concluded that the computed results are in good agreement with the published results and validate the correctness of the mass and stiffness matrices of the developed FE formulation.

### 4.2. Natural Frequencies of a Functionally Graded Nonrotating Simply Supported Shaft

The dimensionless natural frequencies $\bar{\omega }\;({\bar{\omega }}^{4}=\rho_{ss}AL^{4}\omega^{2}/E_{ss}I)$ were obtained for the nonrotating simply supported functionally graded shaft (SS/ZrO_2_) with the material properties given in Table 1 for different modes and power law indices k. The obtained results were compared with the results available in the literature [23] and are tabulated in Table 3. The computed results are in good agreement with the published results; hence, the FG formulations developed in the present work are accurate and can be used for further dynamic analysis.

## 5. Results and Discussion

An FG shaft consisting of an outer ceramic-rich layer made up of zirconium dioxide (ZrO_2_) and an inner metal-rich layer made up of stainless steel (SS) was considered. The gradation of materials occurs between these layers based on the power law and their properties are tabulated in Table 1. The shaft was subjected to thermal load and followed a nonlinear temperature distribution along the radial direction as detailed in Section 2.2. The properties of the shaft, disc and the bearings used in the analysis are given in Table 4. The shaft was divided into fifty finite elements and the corroded shaft element(s) were introduced to study the effects of corrosion on the natural frequency of the system by varying the normalised corrosion parameters such as depth (d/R), length (L_c_/L) and position (X_c_/L).

### 5.1. Effect of d/R for Different X_c_/L Values on the Fundamental Natural Frequency of the System

**Figure 5:**
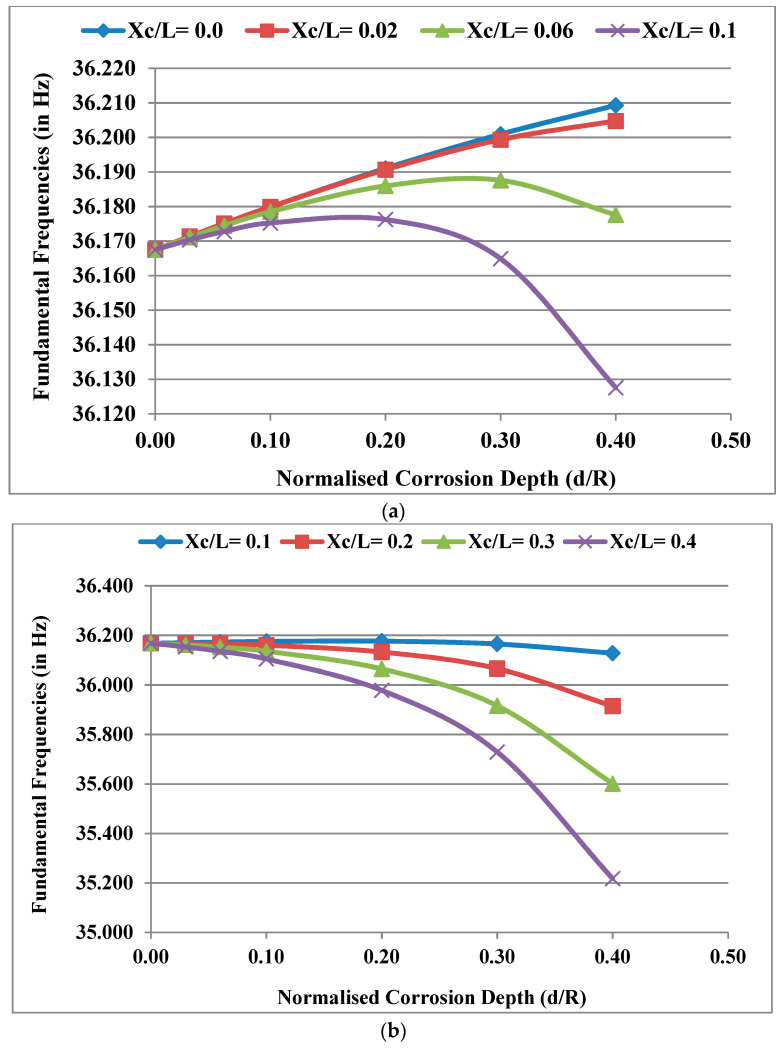
(**a**) Effect of depth (d/R) for different positions (X_c_/L) (≤0.1, corrosion located near the bearings) on the fundamental frequencies of the system; (**b**) effect of d/R for different X_c_/L (0.1 to 0.4), moving away from the bearings and towards the disc on the fundamental frequencies of the system.

Considering the power law index k = 0.5, temperature gradient ΔT = 0 and normalised corrosion length L_c_/L = 0.02, the fundamental frequencies of the system were obtained for different normalised corrosion depths d/R and plotted for different normalised corrosion locations X_c_/L as shown in Figure 5a,b. With an increase in depth (d/R) of the corrosion at the bearings (X_c_/L = 0), the fundamental frequency of the system increases. As the position (X_c_/L) of the corrosion is further away from the bearings as in the case of X_c_/L = 0.1 in Figure 5a, the rate of increase in the frequency decreases and the frequency starts to decrease after reaching a peak. As the corrosion position moves near the disc, the increase in frequency is not noticed and the frequency only decreases with an increasing negative slope as the corrosion depth (d/R) increases, which is clear from Figure 5b. The reason behind the phenomenon is that corrosion results in the loss of mass and a decrease in stiffness, which affects the natural frequencies of the system ($\omega^{2}=k/m)$. The decrease in stiffness of the corroded rotor system will not occur if the corrosion takes place near the bearings due to the significant bearing stiffness. Hence, the effect of a decrease in the stiffness of the rotor system due to an increase in corrosion depth (d/R) is initially insignificant near the bearings compared to the effect of the loss of mass due to corrosion. As a result, the fundamental frequency of the system increases initially due to corrosion before it decreases. However, as the position of corroded shaft elements moves away from the bearings, the effect of a decrease in stiffness due to corrosion becomes more significant than the effect of the loss of mass. As a result, the fundamental frequency of the system decreases due to corrosion.

### 5.2. Effect of d/R for Different L_c_/L Values on the Fundamental Natural Frequency of the System

**Figure 6:**
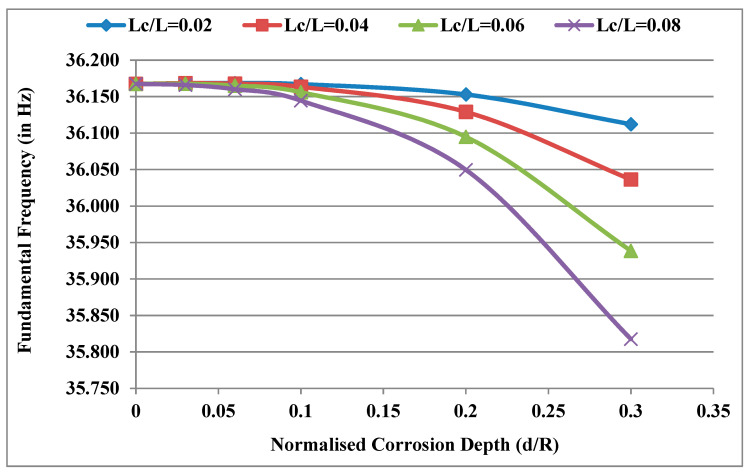
Effect of d/R for different lengths (L_c_/L) on the fundamental frequencies of the system.

Considering the power law index k = 0.5, temperature gradient ΔT = 0 and normalised corrosion position X_c_/L = 0.16, the fundamental frequencies of the system were obtained for different normalised corrosion depths d/R and plotted for different normalised corrosion lengths L_c_/L as shown in Figure 6. It was observed that the rate of decrease in frequency with the increase in corrosion depth increases with the increase in corrosion length. At a constant corrosion depth, the frequency decreases with the increase in corrosion length. The rate of decrease in frequency with the increase in corrosion length increases with the increase in corrosion depth. This is because the stiffness decreases with the increase in corrosion length and depth, and the effect of the decrease in stiffness increases as the length of corrosion increases in the direction away from the bearings and near the disc.

### 5.3. Effect of X_c_/L for Different d/R Values on the Fundamental Natural Frequency of the System

**Figure 7:**
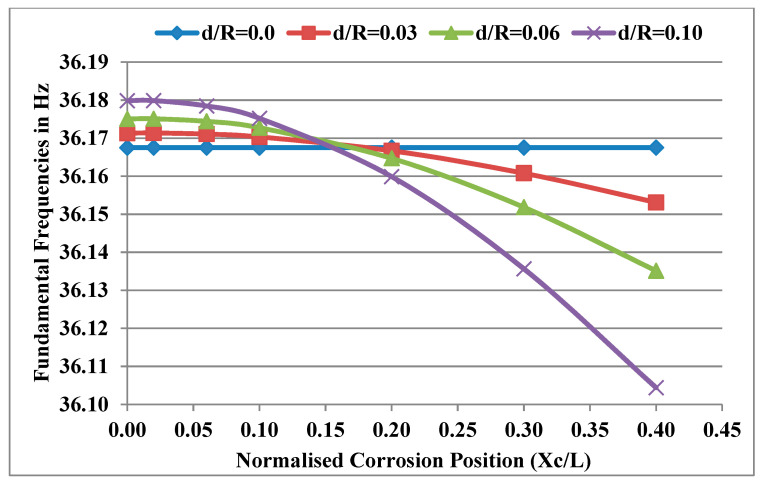
Effect of X_c_/L for different d/R on the fundamental frequencies of the FG rotor system.

Considering the power law index k = 0.5, temperature gradient ΔT = 0 and normalised corrosion length L_c_/L = 0.02, the fundamental natural frequencies of the system were obtained for different normalised corrosion positions and were plotted for different normalised corrosion depths as shown in Figure 7. It was observed that the frequencies decrease with the increase in X_c_/L (corrosion moving away from the bearings) for the same corrosion depth. This is because the effect of a decrease in stiffness due to corrosion becomes more significant than the loss of mass as the corrosion position moves away from the bearings. It was observed that the natural frequencies of the corroded rotor systems are higher than those of the uncorroded rotor systems when corrosion occurs near the bearing due to the predominant loss of mass. For X_c_/L, this is in the range of 0 to 0.16 and is lower when the corrosion causes a predominant decrease in stiffness. The reasons are explained in greater detail in Section 5.1. An important phenomenon can be observed from Figure 7 where all the curves approximately meet at the position X_c_/L = 0.16. This means that the natural frequencies of the corroded rotor system near X_c_/L = 0.16 are unaffected despite deep corrosion defects. At this position, the natural frequency of the corroded rotor system is equal to the natural frequency of the uncorroded rotor system. The reason for this effect is that the loss of mass balances the stiffness reduction of the FG rotor system at X_c_/L = 0.16.

### 5.4. Effect of d/R for Different k Values on the Fundamental Natural Frequency of the System

**Figure 8:**
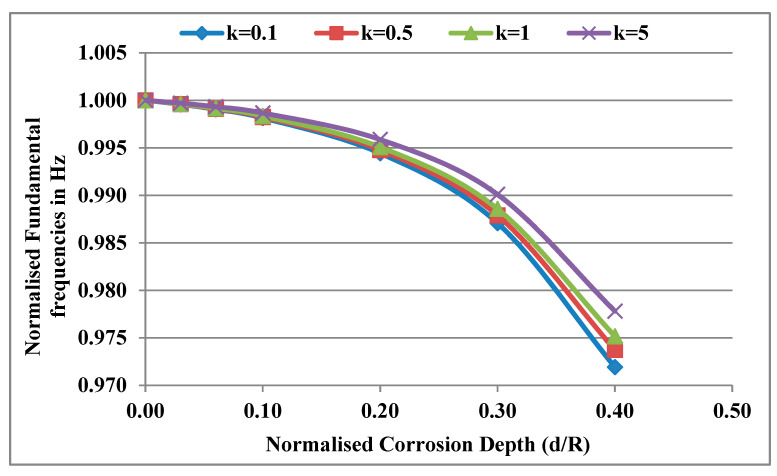
Effect of d/R for different power law indices (k) on the fundamental frequencies of the FG rotor system.

Considering a temperature gradient ΔT = 0 and normalised corrosion length L_c_/L = 0.02, the normalised fundamental frequencies ${\bar{f}}_{c,k}=\;f_{c,k}/f_{uc,k}$ (subscripts *uc* and *c* are uncorroded and corroded, respectively) are computed for different corrosion depths and power law indices. Figure 8 shows the variation of normalised frequencies with the corrosion depth for different power law indices. It was observed that the normalised frequencies decrease with the increase in d/R for any power law index. The rate of decrease of these normalised frequencies was found to decrease with the increase in the k value. The Young’s modulus of the shaft increases from the outer surface to the inner surface with a decreasing slope for higher values of k and with an increasing slope for lower values of k. Therefore, the stiffness of the outermost part of the shaft compared to the innermost part decreases with an increase in the k value. As a result, the percentage reduction of stiffness due to corrosion decreases and the rate of decrease of normalised frequencies decreases with an increase in the k value.

### 5.5. Effect of ΔT and d/R Values at Different X_c_/L on the Fundamental Natural Frequency of the System

**Figure 9:**
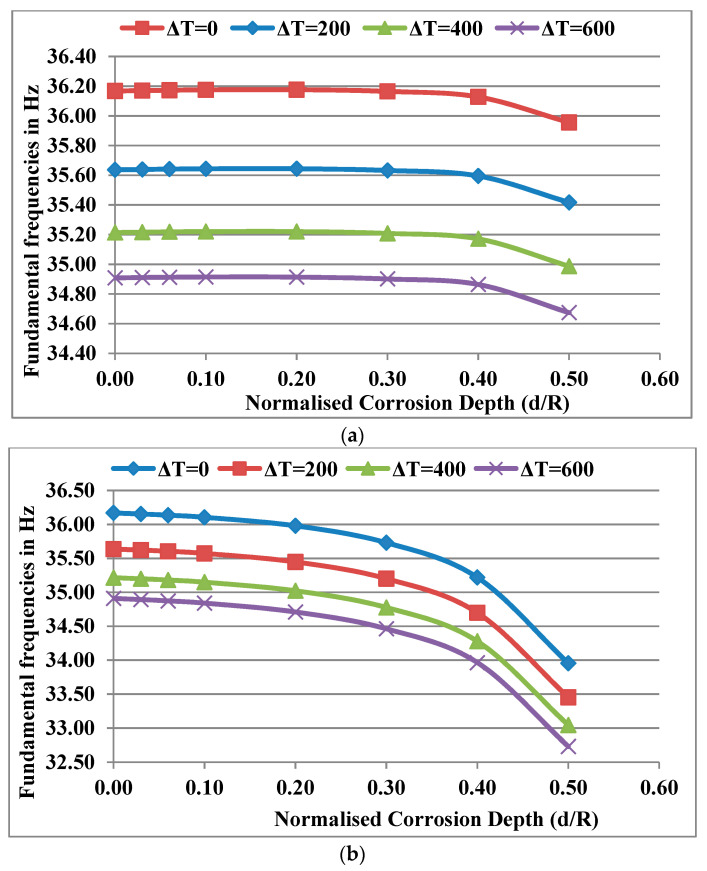
(**a**) Effect of d/R for different ΔT values on the fundamental frequencies of the corroded rotor system for k = 0.5, X_c_/L = 0.1 and L_c_/L = 0.02; (**b**) effect of d/R for different ΔT values on the fundamental frequencies of the corroded rotor system for k = 0.5, X_c_/L = 0.4 and L_c_/L = 0.02.

Considering the power law index k = 0.5, a normalised corrosion position near the bearing X_c_/L = 0.1 and normalised corrosion length L_c_/L = 0.02, the fundamental frequencies obtained for different normalised corrosion depths (d/R) were plotted for different temperature gradients ΔT as shown in in Figure 9a. The frequencies decrease with an increase in d/R at any particular temperature gradient and the rate of decrease in frequencies for different ΔT curves remains the same at any value of d/R. As the temperature gradient increases, the frequencies decrease as expected. Figure 9b shows the variation of frequencies when corrosion occurs near the disc at X_c_/L = 0.4. It was noticed that the rate of decrease in frequencies is comparatively higher as the depth of corrosion (d/R) increases. The variation of frequencies due to corrosion at lower values of X_c_/L (corrosion position near bearings) is due to the predominant effect of the loss of mass, which is not influenced by ΔT. The mass is considered to be independent of temperature. However, at higher values of X_c_/L (for corrosion position away from the bearings and near the disc), the variation in frequencies is due to the predominant decrease in stiffness. The effect of corrosion, which is influenced by ΔT as the stiffness, depends on the temperature-dependent Young’s modulus.

### 5.6. Effect of d/R and X_c_/L on the Change in Whirl Frequencies of the System Due to Corrosion

**Figure 10:**
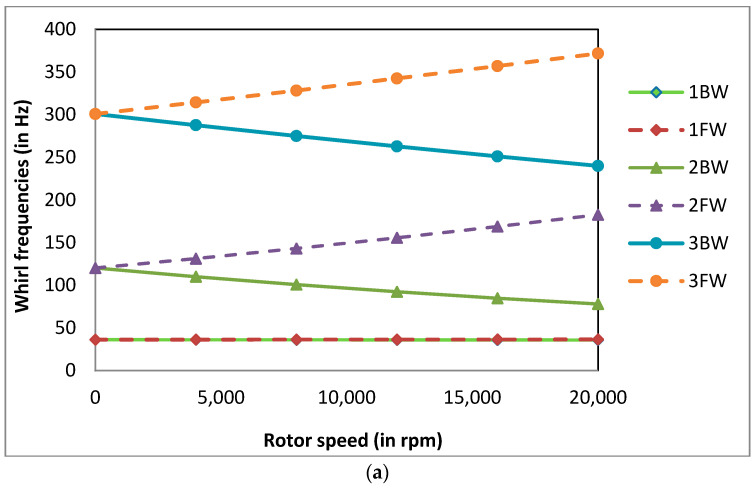

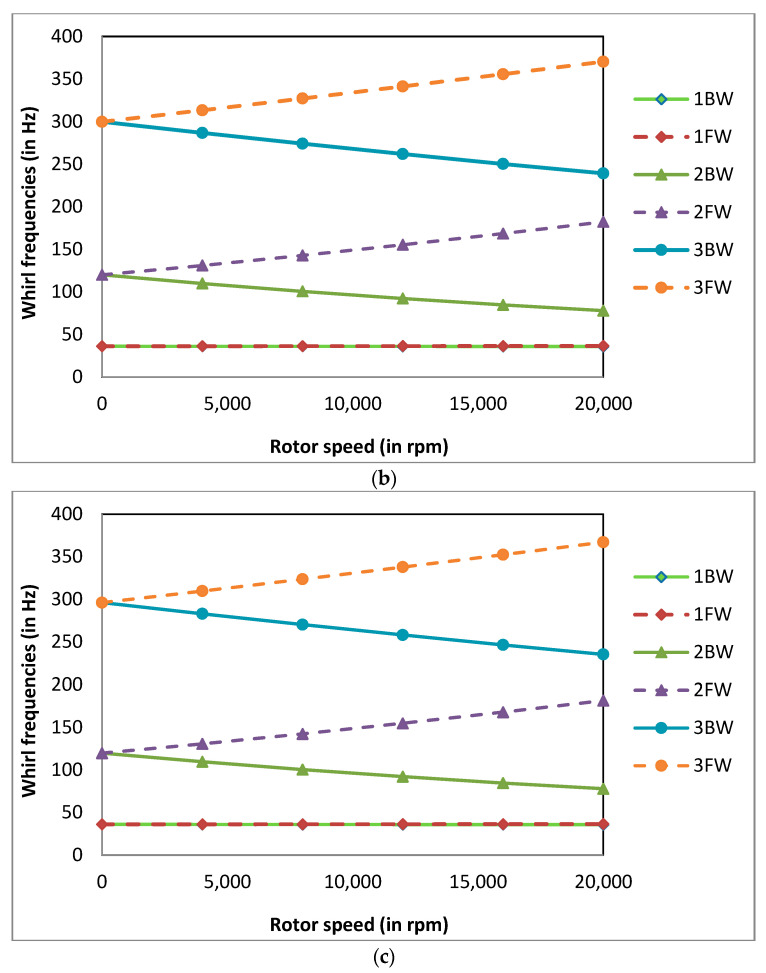
(**a**) Campbell diagram for the FG rotor-bearing system with a corrosion defect at X_c_/L = 0, d/R = 0.1, k = 0.5 and ΔT = 0 K; (**b**) Campbell diagram for the FG rotor-bearing system with a corrosion defect at X_c_/L = 0.1, d/R = 0.1, k= 0.5 and ΔT = 0 K; (**c**) Campbell diagram for the FG rotor-bearing system with a corrosion defect at X_c_/L = 0.4, d/R = 0.1, k = 0.5 and ΔT = 0 K.

The whirl frequencies at Ω = 4000 rpm were calculated for different corrosion depths at different corrosion positions for the power law index k = 0.5, temperature gradient ΔT = 0 K and normalised corrosion length L_c_/L = 0.02. The whirl frequencies are tabulated in Table 5. At lower values of X_c_/L (for corrosion near the bearings), there is an initial increase in the whirl frequencies due to corrosion as the d/R values increase, which is not observed as the X_c_/L value increases (as corrosion position moves away from the bearings) for the same reasons mentioned earlier for natural frequencies. The percentage increase in the whirl frequencies $\;[(\omega_{uc}-\omega_{c})/\omega_{uc}]\times 100\;$ due to the presence of corrosion decreasing for higher values of X_c_/L as the corrosion position moves away from the bearings. As the X_c_/L value increases further (as corrosion position nears the disc), there is no increase in whirl frequencies. The whirl frequencies start to decrease and the percentage decrease in whirl frequencies $[(\omega_{c}-\omega_{uc})/\omega_{uc}]\times 100$ increases. Here, the subscripts *c* and *uc* denote corroded and uncorroded systems, and *ω* denotes the whirl frequencies of the system. Therefore, it is necessary to take precautions to prevent corrosion near the disc rather than near the bearings. The Campbell diagrams at different values of X_c_/L for a normalised corrosion depth d/R = 0.1 are shown in Figure 10a–c. The Campbell diagrams obtained for different corrosion positions are similar. It can be observed from the Campbell diagrams that the split between 1FW and 1BW is negligible compared to 2BW and 2FW.

### 5.7. Effect of Temperature Gradients, ΔT on the Change in Whirl Frequencies of the System Due to Corrosion

**Figure 11:**
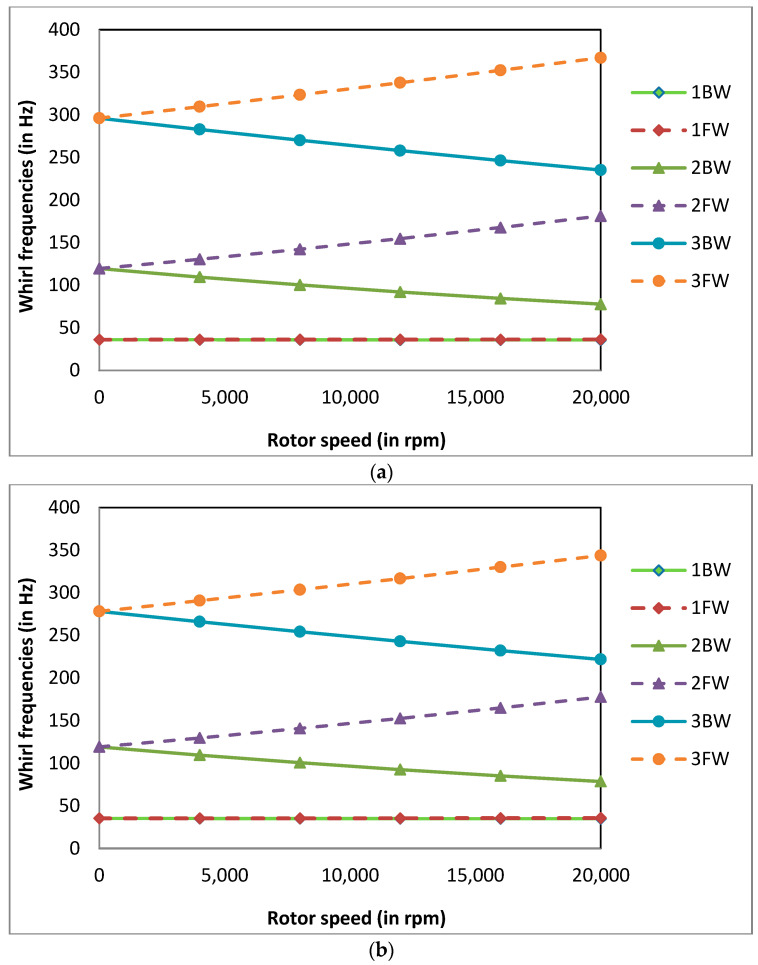

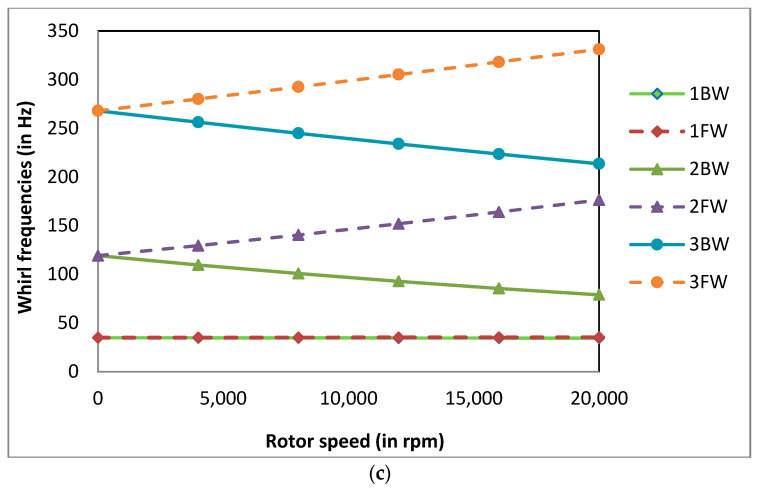
(**a**) Campbell diagram for the FG rotor-bearing system with a corrosion defect at X_c_/L = 0.4, d/R = 0.1, k = 0.5 and ΔT = 0K; (**b**) Campbell diagram for the FG rotor-bearing system with a corrosion defect at X_c_/L = 0.4, d/R = 0.1, k = 0.5 and ΔT = 300K; (**c**) Campbell diagram for the FG rotor-bearing system with a corrosion defect at X_c_/L = 0.4, d/R = 0.1, k = 0.5 and ΔT = 600 K.

The whirl frequencies at Ω = 4000 rpm were calculated for FG rotor-bearing systems with and without corrosion for different temperature gradients of the shaft with the power law index k = 0.5, normalised corrosion depth d/R = 0.1 and normalised corrosion length L_c_/L = 0.02. The whirl frequencies are tabulated in Table 6. It was observed that the whirl frequencies of the rotor system decrease with the increase in temperature gradient of the shaft and decrease due to the presence of corrosion in the rotor system. The percentage decrease in 1BW and 1FW whirl frequency of the rotor system due to the presence of corrosion decreases as the thermal gradient increases. The Campbell diagrams for different values of ΔT at a normalised corrosion depth d/R = 0.1 is shown in Figure 11a–c. The Campbell diagrams obtained for different thermal gradients are similar, therefore they are not plotted here. It can be observed from Campbell diagrams that the split between 1FW and 1BW is negligible compared to 2BW and 2FW.

## 6. Conclusions

The natural and whirl frequency analysis of a functionally graded rotor-bearing system with a corrosion defect was performed using the finite element method to study the effects of corrosion. An FG shaft (SS-ZrO_2_) consisting of an outer ceramic-rich layer and inner metal-rich layer was considered, and the stiffness and mass matrices for the FG shaft element with corrosion was derived. A Python FE code was developed for computing the natural and whirl frequencies of the corroded rotor system. A parametric study was carried out to study the effect of corrosion parameters on the natural and whirl frequencies of the rotor system for different power law indices under a thermal environment. The following important conclusions are drawn from the analysis.

1. The two main effects of corrosion are a loss of mass and decrease in stiffness, which affect the natural frequencies of an FG rotor system. The former results in the increase of natural frequencies and the latter results in the decrease of natural frequencies when only one occurs. However, due to corrosion, both occur simultaneously. An FE modelling was presented to reflect the effects of corrosion in the natural and whirl frequencies of an FG rotor system.
2. If corrosion occurs near the bearings, the effect of a decrease in the stiffness of the rotor system is negligible due to the stiffness offered by the bearings, compared to the effect of the loss of mass of natural frequencies. If corrosion occurs near the disc, the effect of the loss of mass is negligible, compared to the effect of a decrease in the stiffness on natural frequencies.
3. If the depth of corrosion near the bearings increases, the fundamental frequency and whirl frequencies of the rotor system increase due to an increase in the loss of mass. At positions of corrosion slightly away from the bearings, the frequencies start to decrease after attaining a peak with the increase in corrosion depth. When the position of the corrosion nears the disc, the frequencies decrease with the increase in corrosion depth.
4. The rate of decrease in frequency with the increase in corrosion depth increases as the corrosion length increases. At a constant corrosion depth, the frequency decreases with the increase in corrosion length. The rate of decrease in frequency with the increase in corrosion length increases with the increase in corrosion depth.
5. The effect of corrosion decreases for higher values of power law indices. The rate of decrease in fundamental frequency with the increase in corrosion depth decreases for higher values of power law indices.
6. As the ΔT value increases, the rate of decrease in fundamental frequency with the increase in corrosion depth decreases if the corrosion position is near the disc; however, it remains unaffected at corrosion positions away from the disc and near the bearings. The percentage decrease in the 1BW and 1FW whirl frequencies of the rotor system due to the presence of corrosion decreases as the thermal gradient increases.

Since corrosion affects the dynamic characteristics of the rotor systems, it is essential to accurately model and predict dynamic behaviour. The results in the present work would be useful for the research and professional community to give more insight into the effect of corrosion on natural and whirl frequencies of the rotor system under thermal loading.

## Figures and Tables

**Table 1:** Temperature coefficients of material properties.

Properties	Material	*P*_0_	*P*_−1_	*P*_1_	*P*_2_	*P*_3_
E(Pa)	Steel	201.04 × 10^9^	0	3.079 × 10^−4^	−6.534 × 10^−7^	0
	ZrO_2_	244.27 × 10^9^	0	−1.371 × 10^−3^	1.214 × 10^−6^	−3.681 × 10^−10^
V	Steel	0.3262	0	−2.002 × 10^−4^	3.797 × 10^−7^	0
	ZrO_2_	0.2882	0	1.133 × 10^−4^	0	0
K(W/m K)	Steel	15.739	0	−1.264 × 10^−3^	2.092 × 10^−6^	−7.223 × 10^−10^
	ZrO_2_	1.700	0	1.276 × 10^−4^	6.648 × 10^−8^	0

**Table 2:** Natural frequencies of a homogeneous nonrotating simply supported shaft.

Modes	SR	Present	Nelson [29]	Gayen [23]
**1st**	0.02	3.1297	3.1313	3.1298
	0.04	3.0597	3.1017	3.0598
	0.06	3.0438	3.0561	3.0440
	0.08	2.9795	2.9989	2.9797
	0.10	2.9078	2.9343	2.9080
**2nd**	0.02	6.1959	6.2074	6.1960
	0.04	5.9708	6.0079	5.9711
	0.06	5.6840	5.7482	5.6846
	0.08	5.3886	5.4752	5.3894
	0.10	5.1092	5.2126	5.1101

**Table 3:** Natural frequencies for a nonrotating simply supported functionally graded (FG) shaft.

Modes	k = 0.5		k = 1		k = 5	
	Present	Gayen [23]	Present	Gayen [23]	Present	Gayen [23]
**1st**	3.2013	3.2059	3.1763	3.1859	3.1311	3.1505
**2nd**	3.2013	3.2059	3.1763	3.1859	3.1311	3.1505
**3rd**	6.3360	6.3455	6.2872	6.3059	6.1976	6.2356
**4th**	6.3360	6.3455	6.2872	6.3059	6.1976	6.2356
**5th**	9.3598	9.3684	9.2889	9.3096	9.1563	9.2054
**6th**	9.3598	9.3684	9.2889	9.3096	9.1563	9.2054

**Table 4:** The specifications of the disc, shaft and bearings.

**Shaft**	
Length (L)	0.5 m
Radius (R)	0.01 m
**Disc**	
Mass (m_d_)	2 kg
Diametric moment of inertia (I_d_)	0.012 kg-m^2^
Polar moment of inertia (I_p_)	0.024 kg-m^2^
**Bearings**	
Stiffness (K_b_)	10^5^ N/m
Damping constant (C_b_)	100 Ns/m

**Table 5:** Effect of X_c_/L values on change in whirl frequencies due to varying d/R values.

Modes	X_c_/L = 0			X_c_/L = 0.1			X_c_/L = 0.4		
	d/R = 0	d/R = 0.06	d/R = 0.1	d/R = 0	d/R = 0.06	d/R = 0.1	d/R = 0	d/R = 0.06	d/R = 0.1
**1BW**	36.101	36.110	36.115	36.101	36.107	36.110	36.101	36.068	36.036
**1FW**	36.232	36.239	36.243	36.232	36.237	36.239	36.232	36.201	36.171
**2BW**	109.555	109.868	110.063	109.555	109.767	109.896	109.555	109.605	109.627
**2FW**	130.665	131.019	131.253	130.665	130.871	131.009	130.665	130.619	130.599
**3BW**	285.645	286.894	287.681	285.645	286.300	286.696	285.645	284.242	283.038
**3FW**	312.478	313.600	314.359	312.478	312.929	313.246	312.478	310.989	309.737

**Table 6:** Effect of ΔT values on change in whirl frequencies due to corrosion.

Modes	Uncorroded			Corroded, d/R = 0.1, X_c_/L = 0.4		
	ΔT = 0	ΔT = 300	ΔT = 600	ΔT = 0	ΔT = 300	ΔT = 600
**1BW**	36.1013	35.3310	34.8194	36.0363	35.2641	34.7481
**1FW**	36.2322	35.4881	34.9965	36.1710	35.4253	34.9295
**2BW**	109.5550	109.6233	109.5474	109.6271	109.6892	109.6103
**2FW**	130.6648	129.8898	129.4972	130.5992	129.8302	129.4390
**3BW**	285.6454	268.1636	258.2488	283.0379	266.0151	256.2645
**3FW**	312.4778	292.9727	282.2034	309.7369	290.7041	280.1026
